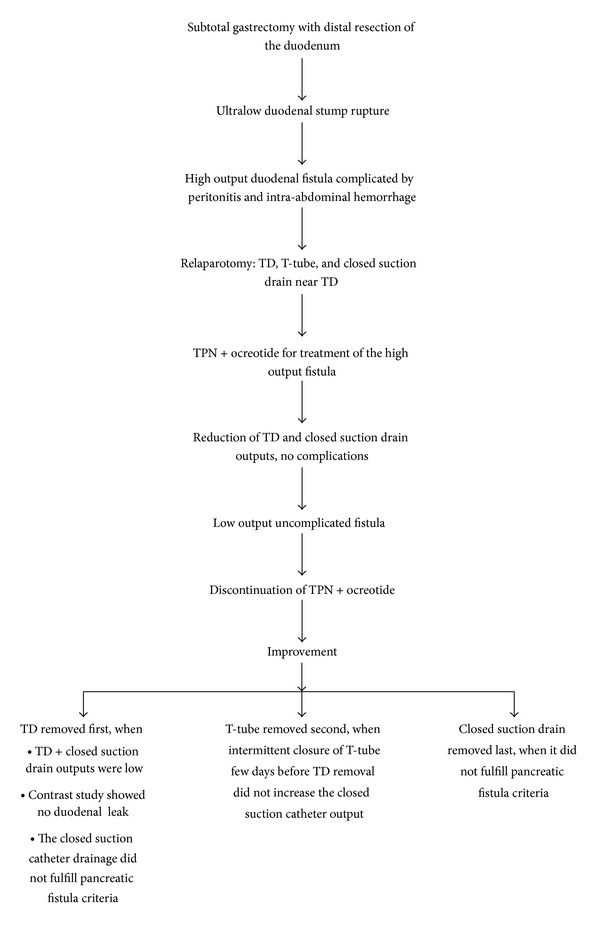# Early Rupture of an Ultralow Duodenal Stump after Extended Surgery for Gastric Cancer with Duodenal Invasion Managed by Tube Duodenostomy and Cholangiostomy

**DOI:** 10.1155/2013/430295

**Published:** 2013-09-18

**Authors:** Konstantinos Blouhos, Konstantinos A. Boulas, Anna Konstantinidou, Ilias I. Salpigktidis, Stavroula P. Katsaouni, Konstantinos Ioannidis, Anestis Hatzigeorgiadis

**Affiliations:** Department of General Surgery, General Hospital of Drama, End of Hippokratous Street, 66100 Drama, Greece

## Abstract

When dealing with gastric cancer with duodenal invasion, gastrectomy with distal resection of the duodenum is necessary to achieve negative distal margin. However, rupture of an ultralow duodenal stump necessitates advanced surgical skills and close postoperative observation. The present study reports a case of an early duodenal stump rupture after subtotal gastrectomy with resection of the whole first part of the duodenum, complete omentectomy, bursectomy, and D2+ lymphadenectomy performed for a pT3pN2pM1 (+ number 13 lymph nodes) adenocarcinoma of the antrum. Duodenal stump rupture was managed successfully by end tube duodenostomy, without omental patching, and tube cholangiostomy. Close assessment of clinical, physical, and radiological signs, output volume, and enzyme concentration of the tube duodenostomy, T-tube, and closed suction drain, which was placed near the tube duodenostomy site to drain the leak around the catheter, dictated postoperative management of the external duodenal fistula.

## 1. Introduction

When dealing with gastric cancer with duodenal invasion, gastrectomy with distal resection of the duodenum is necessary to achieve negative distal margin; however, closure of an ultralow duodenal stump may be difficult. Duodenal stump closure carries a leak rate of 1–3% and a mortality rate of 0–2% in recent series [1]. Early recognition of stump leakage and prompt surgical drainage are essential to a lowering of mortality and morbidity. Duodenal drainage can be obtained with either tube duodenostomy (TD) along with tube cholangiostomy (TC) or tube duodenocholangiostomy [2]. In parallel with duodenal drainage, biliary diversion, gastric diversion with Roux-en-Y reconstruction, secondary suture of the duodenal leak, although usually not feasible, and close postoperative observation are paramount requirements to provide a consummate approach to duodenal stump dehiscence [3]. 

The present paper describes a case of an ultralow duodenal stump rupture after an extended gastrectomy in a patient with gastric cancer of the antrum and duodenal invasion; the rupture occurred on postoperative day 1, which is an extremely rare event. Following stump rupture, hemoperitoneum occurred due to erosion of the unsheathed transverse mesocolon vessels from duodenal contents. After ligation of the bleeding vessels, duodenal drainage was accomplished by TD and TC. 

## 2. Case Presentation

A 62-year-old male patient presented to our surgical department after observing the passage of black, tarry stools per rectum. Esophagogastroduodenoscopy identified an advanced type 3 60 × 30 mm tumor of the lower part of the stomach and at least 15 mm extension into the duodenum. Endoscopic biopsy revealed a poorly differentiated adenocarcinoma. An abdominal and thoracic CT obtained showing thickening of the gastric wall of the antrum and the first part of the duodenum, absence of serosa invasion, associated regional lymph nodes, and absence of distant metastasis. 

**Figure 1:**
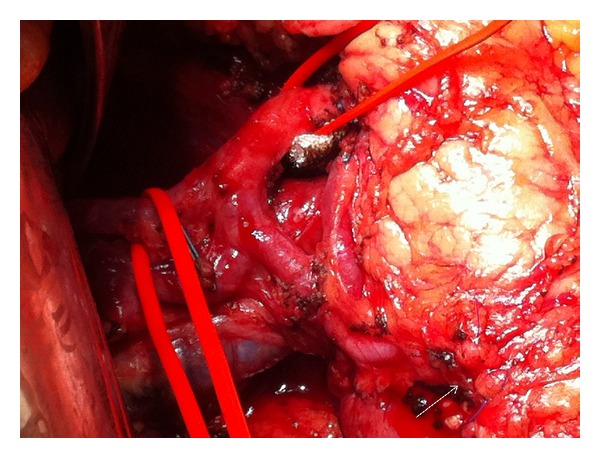
During initial laparotomy, the lateral aspect of the surgical bed after completion of bursectomy and the closed ultralow duodenal stump (arrow) can be seen.

An extended gastrectomy was decided for a resectable cT3cN+cM0 tumor; however, pancreaticoduodenectomy was in mind if a clear distal margin could not be ensured. In our case, extended surgery consisted of (a) distal gastrectomy and at least 45 mm resection of the first part of the duodenum. Frozen section examination of the resection line showed no microinvasion of the carcinoma; consequently, an ultralow duodenal stump was left behind in the surgical bed (Figure 1) and closed after mobilization of 10 mm of the posterior duodenal wall from the pancreas using a 45 mm linear cutting stapler and interrupted seromuscular layer sutures; (b) complete omentectomy and bursectomy; (c) D2 plus number 11d, 12b, 12p, 13, 14v, 16a2, 16b1, 17, and 18 lymphadenectomy; (d) Roux-en-Y gastrojejunostomy in an antecolic way. Histology of the surgical specimen revealed a type 3, 60 × 30 mm, PM0, DM0, R0, CY(0), UL(+), ly(+), v(+), and poorly differentiated solid type pT3pN2pM1 and stage IV adenocarcinoma with 6/68 metastatic lymph node ratio [4]. The tumor was characterized as M1 due to involvement of number 13 nodes (1/3 metastatic ratio).

**Figure 2:**
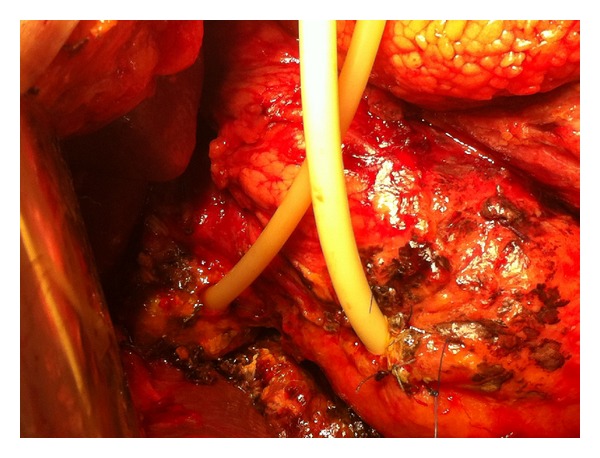
During relaparotomy, the ruptured ultralow duodenal stump was managed by tube duodenostomy and cholangiostomy.

The patient was convalescing satisfactory; until suddenly on postoperative day 1 severe pain, abdominal rigidity, and fever developed. Bile-stained fluid was observed at the drain near the duodenal stump. Few hours after the onset of pain the patient exhibited a shock-like state and became hemodynamically unstable. Red blood was withdrawn from the drain near the gastrojejunal anastomosis. Relaparotomy was performed; dehiscence of the corner of the medial wall of the duodenal stump and hemoperitoneum due to erosion of vessels from the duodenal content at the left side of the transverse mesocolon, which left skeletalized due to bursectomy, were observed. After thorough peritoneal lavage and ligation of the bleeding mesenteric vessels, duodenal drainage and biliary diversion were performed (Figure 2). A 22 French Foley catheter was introduced through the open end of the duodenal stump. A pursestring of 3-0 absorbable suture was placed near the open edge of the duodenum and tied so that it gently held the tube in place. A second pursestring of 3-0 nonabsorbable suture was placed around the tube in the seromuscular layer and tied so that the first pursestring was invaginated into the lumen of the duodenum. Unfortunately, due to complete omentectomy, an omental pedicle was not available to secure the junction of the tube and the duodenum. The tube was then brought out through the abdominal wall leaving the intraperitoneal portion of the tube as short as possible. A closed suction drain was left near TD site to drain the leak around the catheter, and T-tube drainage of the common bile duct was added.

**Figure 3:**
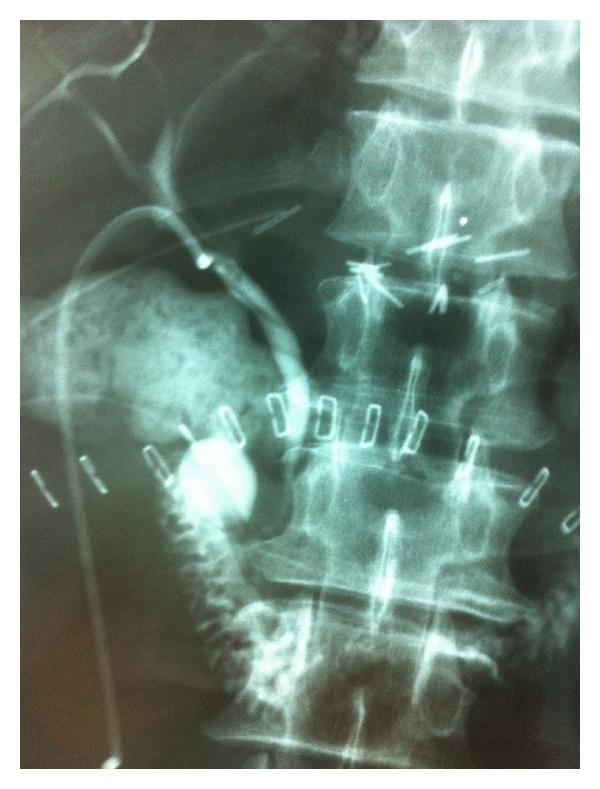
Contrast graph through tube duodenostomy. The tube duodenostomy, the closed suction drain, and the T-tube can be seen.

**Figure 4:**
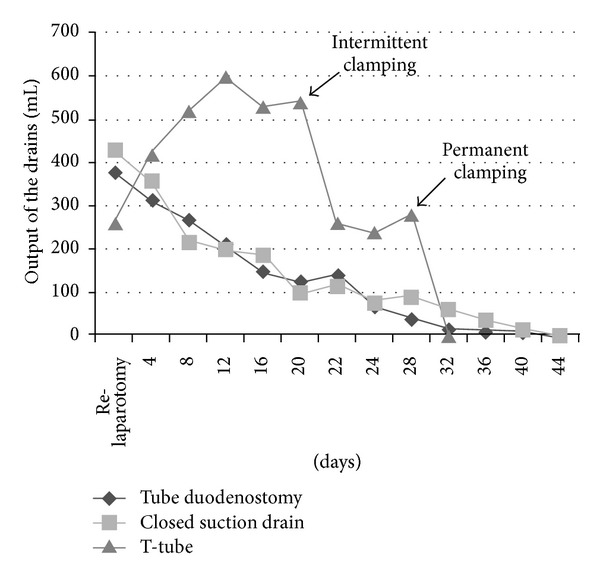
Comparison of mean daily outputs of the tube duodenostomy, T-tube, and closed suction drain near the tube duodenostomy. The lack of omental patch is depicted by the high output of the closed suction drain which reflects duodenal leak from the side of the tube duodenostomy.

After the primary postoperative period, total parenteral nutrition and ocreotide at a dose of 100 mcg subcutaneously 3 times per day were administered; enteral diet was instituted on postoperative day 15. Assessment of clinical, physical, and radiological signs (Figure 3), output volume, and enzyme concentration of TD, T-tube, and closed suction drain (Figure 4) was employed in the overall management of the duodenocutaneous fistula (Figure 5). During hospitalization, the patient did not develop symptoms and signs of peritonitis or sepsis. The TD was removed on postoperative day 28, when (a) the output of the TD was minimum; (b) a contrast study did not reveal leak around the TD catheter; and (c) the closed suction catheter drainage did not fulfill pancreatic fistula criteria regarding output and amylase concentration [5]. The T-tube catheter was removed on postoperative day 32 with intermittent clamping 7 days before TD removal and permanent clamping after TD removal, when despite clamping, the output of the closed suction drain remained low. Finally, on postoperative day 36, the closed drain catheter was removed, and the patient was discharged home; the external duodenal fistula ceased spontaneously on postoperative day 45.

## 3. Discussion

Gastric cancer with duodenal invasion has been reported with an incidence of 15–40% [6]. Kakeji et al. analyzed 95 patients with duodenal invasion by gastric cancer and found that tumor spread into the duodenum was limited to within 2 cm in 76% of the patients and to within 3 cm in 81% of the patients. Therefore, he suggested gastrectomy with resection of 3-4 cm of the duodenum for patients with advanced gastric cancer and duodenal invasion [7]. However, closure of an ultralow duodenal stump may be difficult, and today's surgeons should be familiar with the procedures that have been developed for dealing with this problem, such as standard duodenal closures, Nissen's closure, Bancroft's closure, and primary TD [8]. However, when facing duodenal stump rupture, there it is one way to provide adequate duodenal drainage which isby secondary TD.

The most successful method of managing duodenal stump rupture has been TD; however, published series about TD have either few patients or insufficient information related to indication, technique, and postoperative care [9]. When performing TD for duodenal stump rupture, questions about technical details arise: (a) end or lateral duodenostomy? We chose end duodenostomy in an effort to create a controlled duodenal fistula; lateral duodenostomy is primarily used for intraluminal decompression and is not employed when technical factors prevent adequate closure of the duodenal stump [10]; (b) TD with or without TC? By draining the common bile duct, we accomplish to decrease the output of the TD by draining out the bile from the upper duodenum, we managed to decrease duodenal leak from the side of the TD, which was expressed by decreased closed suction drain output, and we gain time for relaxation of partial obstruction from edema in the distal common bile duct caused by the sutures placed around the tube in the seromuscular layer of the duodenum, as a result of the close anatomic relationship [11]; (c) what about TD without omental patch? In our case, a viable omental pedicle was not secured around the junction of the tube and the duodenum due to complete omentectomy; consequently, the output of the closed suction drain was higher and the time interval for closure of the external duodenal fistula was longer than previously reported by other studies in the literature [12].

What is interesting in our case is that duodenal stump rupture occurred on postoperative day 1. Technical failure such as malfunction of the linear stapler or overzealous closure and avascularization of the distal duodenal stump due to the radicality of the procedure that we performed are implicated for duodenal stump rupture [13]. Avascularization of the distal duodenal stump can be attributed to (a) duodenal skeletalization due to Kocher maneuver and separation of the gastrocolic omentum from the first and second parts of the duodenum by ligating small vessels which pass between them; (b) bursectomy; (c) although the trunk of the gastroduodenal artery preserved, other more peripheral branches, like the supraduodenal, retroduodenal, right gastroepiploic, and anterior superior pancreaticoduodenal arteries were divided; (d) wide dissection of the posterior and anterior surface of the pancreatic head (numbers 13 and 17 lymph nodes dissection), which disturbed the already deteriorated blood supply to the second portion of the duodenum. 

Another remarkable fact is intra-abdominal hemorrhage due to erosion of vessels from the duodenal content at the left side of the transverse mesocolon whichoriginated few hours after duodenal stump rupture. Bursectomy definitely played a role as the superior layer of the peritoneum of the transverse mesocolon was dissected along with the posterior layer of the peritoneum of the greater omentum leaving the anterior leaf of the transverse mesocolon unsheathed. Another point we should highlight in our case is that histopathology revealed involvement of number 13 lymph nodes. The Japanese gastric cancer treatment guidelines suggest dissection of number 13 lymph nodes for tumors invading the duodenum [14]; indeed, our decision changed adjuvant therapy strategy as the disease was classified as pM1 and prevented early obstructive jaundice resulting from retropancreatic nodes metastasis. 

In conclusion, rupture of the ultralow duodenal stump due to extensive avascularization of the second part of the duodenum and intra-abdominal hemorrhage due to erosion of small vessels from the unsheathed transverse mesocolon are potential complications after extended surgery for gastric cancer with duodenal invasion. End TD, even without an omental patch, and TC along with close postoperative observation are a successful method to manage the leakage. 

## Figures and Tables

**Figure 5:**